# The College of Nursing (Limited by Guarantee)

**Published:** 1920-10-02

**Authors:** 


### The College of Nursing
### (Limited by Guarantee).

The quarterly meeting of the Irish Board was held at 54 Fitzwilliam Square, Dublin, on September 20, Sir John Moore presiding. There were also present: Miss Adams, Miss Chisholm (Dublin); Miss Curtin, Mrs. Mawhinney (Belfast); Miss Michie and Miss Thomas (Dublin).

A resolution of sympathy with the relatives of the late Sir Alexander Dempsey, who was an original and most valued member of the Board, was passed in silence, all standing. A resolution of sympathy with Sir Andrew Horne in his recent bereavement was passed. The results of the annual election were announced, and hon. officers were elected for the session 1920-21.

It was reported that on a referendum taken by the Board on the question of whether nurses would wish to be included in the Hours of Employment Bill under a Special Order, as suggested by the College of Nursing, 95 per cent. of the members voting were in favour of inclusion. The College scheme provides for a fifty-six-hour week for nurses, with adaptations which render its adoption possible both for institution and private nurses without resultant loss of work, or hardship to the public, such as would be the effect of an unconditional forty-eight-hour week. The result of the referendum was reported to have been communicated to the Minister of Labour. With the object of keeping the Board in closer touch with the needs of College members and facilitating the carrying out of its published programme, a resolution was adopted dissolving the existing sub-committees and substituting an Executive and Finance Committee to meet fortnightly. Preliminary arrangements were made for the establishment of Local Centres of the College in Dublin and Belfast. A most encouraging letter from the nurses in the Downpatrick Union Infirmary was read, reporting the final success of their appeal for an increase of salary and ration allowance, and thanking the Irish Board very heartily for all the trouble they had taken on the nurses' behalf in this matter. Other appeals from union-infirmary nurses were considered and dealt with. Other business having been transacted, the meeting adjourned.

### BIRMINGHAM THREE COUNTIES CENTRE.

On September 24 Miss Musson, R.R.C., and the hon. officers of the Centre were "at home" to members and their friends. The invitations bore the magic words "Whist Drive," and were eagerly accepted. The Governors of the General Hospital, Birmingham, kindly lent the Board Room, which, charmingly decorated with pink and white chrysanthemums, suffered a complete metamorphosis.

On September 27 the Lord Mayor of Birmingham (Alderman W. A. Cadbury) called a meeting at the Council House to consider a scheme to raise £10,000 as a thank-offering to nurses for their services during the late war. In the course of his address he reminded those present of the debt of honour they owed to the nursing services.

The Hon. Sir Arthur Stanley, who had travelled specially from London to address the meeting, then spoke of the College of Nursing, its aims, its work, and its accomplishments. He reminded the meeting that it was owing to the grant made by the Government to Miss Florence Nightingale that the first training-school for nurses had been established. He pleaded eloquently for shorter hours, more adequate remuneration, and improved conditions of work. Also he accentuated the need for a hostel and lecture-room for the Birmingham and Three Counties Centre.

The scheme for raising the money—a large Scenic Fair, to be held at the Bingley Hall in the spring—was then outlined by the Organising Secretary, Mrs. Richards, who is editing *A Herald*, in which all news of the Fair may be found, to be published monthly for the next six months. Copies may be obtained from No. 3 New Street. Subscription for the series, 2s.

Mr. Billington spoke of the need for the higher education of nurses, and submitted a resolution, which was passed unanimously, approving the action of the Lord Mayor in calling the meeting, and promising the active support of all present to ensure the success of the undertaking.

